# Preliminary study on pathogenic mechanism of first Chinese family with PNKD

**DOI:** 10.1515/tnsci-2022-0222

**Published:** 2022-06-09

**Authors:** Feng Chen, Shaohui Zhang, Tinghong Liu, Liu Yuan, Yangshuo Wang, Guojun Zhang, Shuli Liang

**Affiliations:** Functional Neurosurgery Department, National Children’s Health Center of China, Beijing Children’s Hospital, Capital Medical University, No. 56, Nanlishi Road, Xicheng District, Beijing, 100045, China; Neurosurgery Department, PLA General Hospital, No. 28 Fuxing Road, Haidian District, Beijing, 100853, China; Functional Neurosurgery Department, Beijing Children’s Hospital, Capital Medical University, National Center for Children’s Health, Beijing, China, No. 56, Nanlishi Road, Xicheng District, Beijing, 100045, China; Key Laboratory of Major Diseases in Children, Ministry of Education, Beijing, China, No. 56, Nanlishi Road, Xicheng District, Beijing, 100045, China

**Keywords:** gene mutation, gene function, paroxysmal non-kinesigenic dystonia, isobaric tags for relative and absolute quantitation

## Abstract

**Background:**

The first Chinese family with paroxysmal non-kinesigenic dystonia (PNKD) was confirmed to harbour a *PNKD* mutation. However, the pathogenic mechanism of the PNKD-causing gene mutation was unclear.

**Methods:**

Wild-type and mutant *PNKD-L* plasmids were prepared and transfected into the C6 cell line to study the distribution and stability of PNKD protein in C6 cells and its effect on the glutathione content. The blood and cerebrospinal fluid (CSF) of 3 PNKD patients and 3 healthy controls were collected. The differentially expressed proteins were identified using isobaric tags for relative and absolute quantitation. Furthermore, Gene Ontology (GO) and Kyoto Encyclopaedia of Genes and Genomes (KEGG) enrichment analyses were performed, and the protein–protein interaction network was constructed.

**Results:**

Wild-type PNKD protein was mainly distributed in the membranes, whereas mutant PNKD protein was distributed throughout the C6 cells. After transfection with mutant *PNKD-L* plasmid, the glutathione content decreased significantly in C6 cells; the stability of the mutant PNKD protein was significantly low. There were 172 and 163 differentially expressed proteins in CSF and plasma, respectively, of PNKD patients and healthy controls. For these proteins, blood microparticle and complex activation (classical pathway) were the common GO enrichment term, and complex and coordination cascade pathway were the common KEGG enrichment pathway. Recombinant mothers against decapentaplegic homolog 4 (SMAD4) was one of the differentially expressed proteins; it exhibited a relationship with the aforementioned enrichment GO terms and KEGG pathway.

**Conclusion:**

PNKD protein was mainly distributed in cell membranes. *PNKD-L* mutation affected subcellular localisation, PNKD protein stability, and glutathione content. SMAD4 was found to be a potential biomarker for PNKD diagnosis.

## 1 Background

Paroxysmal non-kinesigenic dyskinesia (PNKD) is a rare movement disorder that manifests as choreatic/dystonic movements with preserved consciousness during attacks. Primary PNKD is an idiopathic or genetic disorder, whereas secondary PNKD is associated with various neurological and medical conditions [1,2]. PNKD attacks are often related to the consumption of alcohol, coffee, tea, and other irritants. They can also be induced by fatigue, stress, and excitement. Some patients have no clear inducement. The duration of the attack can range from several minutes to hours or more than a day. The attack manifests as various combinations of dystonia, athetosis, and chorea. The frequency of seizures varies from several times a year to several times a day [3,4]. PNKD was first reported by Mount and Reback in 1940 and showed autosomal dominant inheritance. Its pathogenic gene is the *PNKD* gene on 2q35 chromosome [5], and many PNKD cases were confirmed to have been caused by A7V, A9V, and A33P mutations [5,6,7]. *PNKD* is mainly expressed in skeletal muscle and the heart, as well as in the brain, and has at least three shear types: *PNKD-L*, *PNKD-M*, and *PNKD-S* [2,6,7,8]. *PNKD-L* is specifically expressed in the brain [6,7,8].

The pathogenic mechanism of PNKD is not clear. We studied the first Chinese PNKD family in mainland China [9] and carried out preliminary research on the *PNKD* gene function and its pathogenic mechanism.

## 2 Methods

### 2.1 Collection of clinical data and samples from PNKD family

After clinical analysis and gene sequencing, one male patient was diagnosed with PNKD with A7V mutation of the *PNKD* gene. After face-to-face consultation and physical examination of the main members of the proband’s family, the family tree was obtained [9]. The cerebrospinal fluid (CSF) and blood samples of three patients and three healthy controls in this family were collected.


**Ethical approval:** The research related to human use has been complied with all the relevant national regulations, institutional policies and in accordance with the tenets of the Helsinki Declaration, and has been approved by the Ethics Committee of the Fourth Medical Center, PLA General Hospital. All subjects signed the written consent to participate in the study.

### 2.2 Functional test of PNKD-L

#### 2.2.1 Construction of wild-type and mutant PNKD-EGFP expression plasmids

The coding sequence of the wild-type *PNKD-L* gene was synthesised using the whole gene, and a ligation reaction was carried out with ligase and pMD19-T vector overnight at 16°C. Clones were randomly selected and transferred into kanamycin-resistant (80 mg/L) LB medium, cultured overnight at 37°C, and shaken. PCR expansion was carried out with mutant primers and 30 ng wild-type plasmid. The PCR products were digested using KPNL and electro-transferred to *Escherichia coli* (360 V, 1 s); after monoclonal antibody sequencing comparison was performed, the obtained recombinant plasmids were named pEGFP-WT-1 (WT) and pEGFP-MT-1 (MUT).

#### 2.2.2 Transfection of C6 cell line

Human glioma C6 cells (1 × 10^6^) were seeded in a 12-well culture plate (60 mm) and cultured overnight in DMEM solution containing 100 mg/mL streptomycin, 100 U/mL penicillin, 10% foetal bovine serum, and 1 mM sodium pyruvate. The incubator was maintained at 37°C and contained 5% CO_2_. When the cells overgrew (by 60–80%) the wells, Lipofectamine 2000 was used to transfect plasmids (WT and MUT) (Plasmid:Lipofectamine 2000 = 1:2).

#### 2.2.3 Subcellular localisation

After C6 cells were transfected for 24 h, they were transferred to a cell-climbing sheet. The cells were fixed with 95% ethanol for 20 min and washed with PBS for 2 min thrice. After the PBS solution was sucked and discarded, the distribution of PNKD-L in C6 cells was directly observed under a Leica SP5 fluorescence microscope. The excitation and emission wavelengths were 488 and 508 nm, respectively.

#### 2.2.4 Test of glutathione content

The transfected C6 cells were rinsed and lysed in RIPA solution containing protease inhibitor, and the supernatant was obtained after centrifugation and balanced at room temperature for 30 min. According to the kit instructions, the standard configuration was diluted to each concentration gradient. Coenzyme working solution (20 µL), buffer solution (120 µL), and enzyme working solution (20 µL) were added into each standard and sample hole. The samples were incubated at 37°C for 5 min while shaking at 500–1,000 rpm. An aliquot of the sample (20 µL) was added to each well and incubated at 37°C for 10 min; then, 20 µL substrate working solution was added to each well and incubated at room temperature for 10 min. The absorption was measured at 405 nm on a microplate reader. Each sample in three subgroups was analysed.

#### 2.2.5 PNKD protein stability analysis

After transfection of C6 cells for 24 h, CHX treatment was carried out. The cells collected at different timepoints were lysed in RIPA buffer, and protein electrophoresis loading buffer was added in proportion. Freezing at −20°C, incubation at 95°C for 10 min, protein electrophoresis, membrane transfer, and milk sealing were successively performed. After TBST rinsing, EGFP primary antibody and a secondary antibody were added and an ECL colour reaction was performed. Each sample in three subgroups was measured, and the mean value was taken for the *F* test. *P* < 0.05 was defined as a significant difference.

### 2.3 Proteomic analysis

#### 2.3.1 Differentially expressed proteins in CSF and plasma of PNKD patients and controls

CSF routine and biochemical examinations were performed. Proteins were extracted using the ultrafiltration tube method and those with molecular weight over 3 kD were selected. A standard curve was established and the total protein was quantified using the BCA method. Then, SDS-PAGE was carried out. The protein was hydrolysed using trypsin, and 80 μg of peptide segments of each group were labelled according to the instructions of the Isobaric tags for relative and absolute quantitation (iTRAQ) reagent-8 plex multiplex kit (AB SCIEX). The enzymatic peptides were analysed using a combination of high-performance liquid chromatography and mass spectrometry analysis. Proteome discoverer (Thermo) software was used for library identification and quantitative analysis of peptides. The database is Ensembl Homo sapiens.iwgsc 1.0 + popseq.28.pep. The retrieved peptide and spectrum matching was filtered using the Percolator algorithm, with the *q* value less than 1%. The retrieved peptides were combined into proteins using the strict principle of maximum parsimony. Ratios <0.67 or >1.5 (*P*-values ≤ 0.05) were used as the screening criteria for differential proteins. The results were used for bioinformatics analysis.

#### 2.3.2 Gene Ontology (GO) enrichment and Kyoto Encyclopaedia of Genes and Genomes (KEGG) enrichment analysis

Protein numbers were retrieved from UniProt. GO is a structured and controlled vocabulary of terms. The terms are subdivided into three non-overlapping ontologies – Molecular Function (MF), Biological Process (BP), and Cellular Component (CC) – and are used widely for annotating genes and gene products. The KEGG is a knowledge base for systematic analysis of gene functions, linking genomic information with higher order functional information, and is used widely for pathway-related analysis. In this study, the Parent-Child-Intersection method was used for enrichment analysis and the Benjamini-Hochberg procedure was used for multiple test corrections. An adjusted *P* value of < 0.05 was set as the cut-off criterion.

#### 2.3.3 Biological network analysis

The signal transmission networks of differentially expressed proteins and PNKD disease-related proteins in CSF and plasma were constructed by comparing KEGG and STRING databases, respectively, to identify the relationship between differentially expressed proteins and diseases. The construction method employed was as follows: search for the proteins upstream and downstream of differentially expressed proteins and PNKD disease-related proteins (PNKD/ hydroxyglutathione hydrolase/glutathione synthetase) in KEGG and STRING databases, find the protein connections between differentially expressed proteins and disease-related proteins, and draw the connection relationship between the proteins using Cytoscape software.

## 3 Results

### 3.1 Functional abnormality of mutant PNKD

#### 3.1.1 Subcellular localisation and stability of mutant PNKD protein

**Figure 1:**
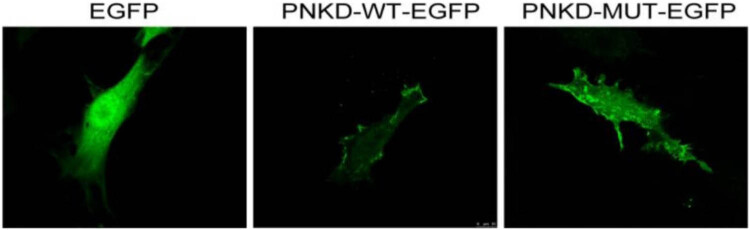
Subcellular localisation of PNKD proteins. This figure includes three fluorescence microscope pictures to show the different localisation behaviours of PNKD proteins in different cells. EGFP shows fluorescence of a control normal C6 cell; PNKD-WT-EGFP shows that PNKD protein localised mainly in membranes of C6 cells transfected with wild-type PNKD plasmids; and PNKD-MT-EGFP shows that PNKD localised in the membrane and cytoplasm of C6 cells transfected with mutant PNKD plasmids. The distribution range of PNKD protein was significantly wider in mutant PNKD cells than that in wild-type PNKD cells.

**Figure 2:**
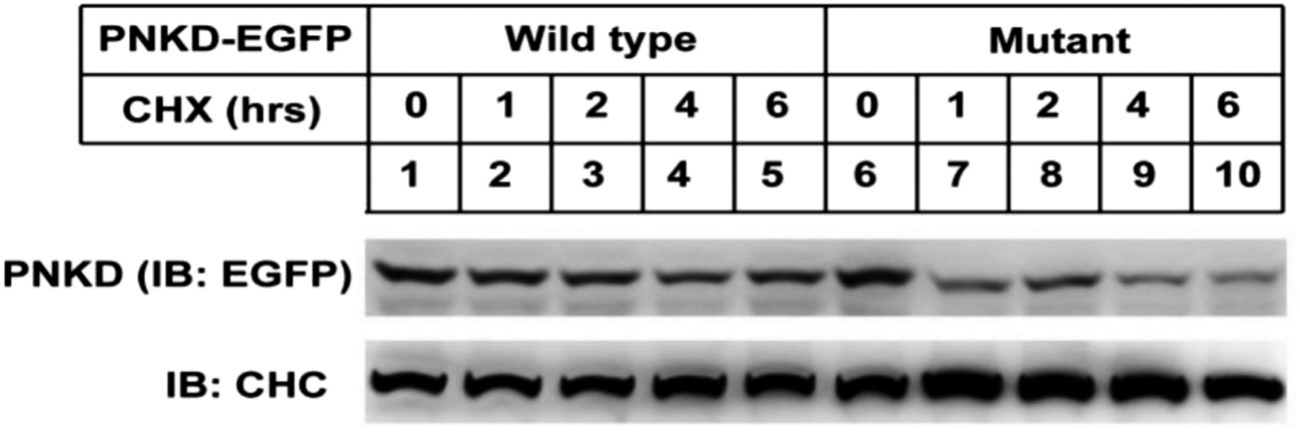
Comparison of stability of wild-type and mutant PNKD proteins at different timepoints. This figure shows that the electrophoresis patterns of the mutant PNKD protein became weaker, whereas the fluorescence of the wild-type PNKD protein was relatively stable for 6 h, indicating the lower stability of the mutant PNKD protein. The electrophoretic bands provided in the picture are complete, but from different gels. The raw pictures of gels are included in the supplementary material.

The sequences of wild-type and mutant *PNKD-EGF* expression plasmids were confirmed to be correct using sequencing and comparison. The wild-type PNKD protein was mainly distributed in the cell membrane, whereas the mutant PNKD protein was distributed throughout the cell (its distribution range was significantly wider than that of the wild-type protein) (Figure 1). Furthermore, the stability of the mutant PNKD protein was significantly lower than that of the wild-type protein in the C6 cell line (Figure 2).

#### 3.1.2 Effect of PNKD mutation on glutathione

**Figure 3:**
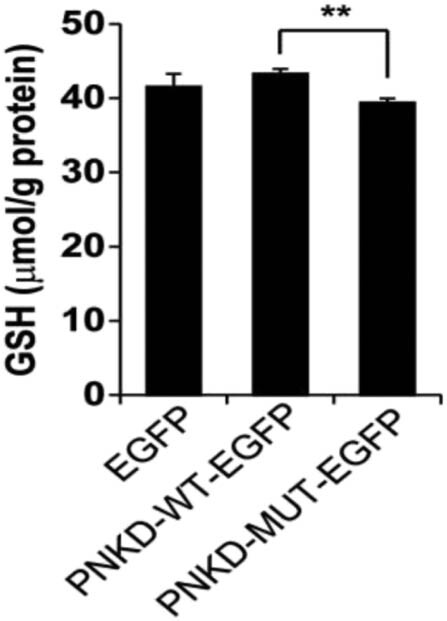
Comparison of glutathione content in C6 cells with wild-type and mutant PNKD (*P < 0.01).

As shown in Figure 2, the glutathione contents in C6 cells transfected with wild-type and mutant plasmids were 43.27 ± 0.67 and 39.39 ± 0.77 µmol/g, respectively, and the contents in cells with *PNKD* mutation were significantly lower than those in cells without mutant *PNKD* (*P* < 0.01) (Figure 3).

### 3.2 Proteomic analysis

#### 3.2.1 Differentially expressed proteins in CSF and plasma

There was no PNKD protein expression in CSF. Significance analysis indicated that the number of significantly differentially expressed proteins in CSF was 172 for healthy controls and PNKD patients. Also, no PNKD protein expression was found in plasma, and there were 163 significantly differentially expressed proteins in plasma of PNKD patients compared to that of healthy controls.

#### 3.2.2 Enrichment analysis of functions and signal pathways of differentially expressed proteins in CSF and plasma

**Figure 4:**
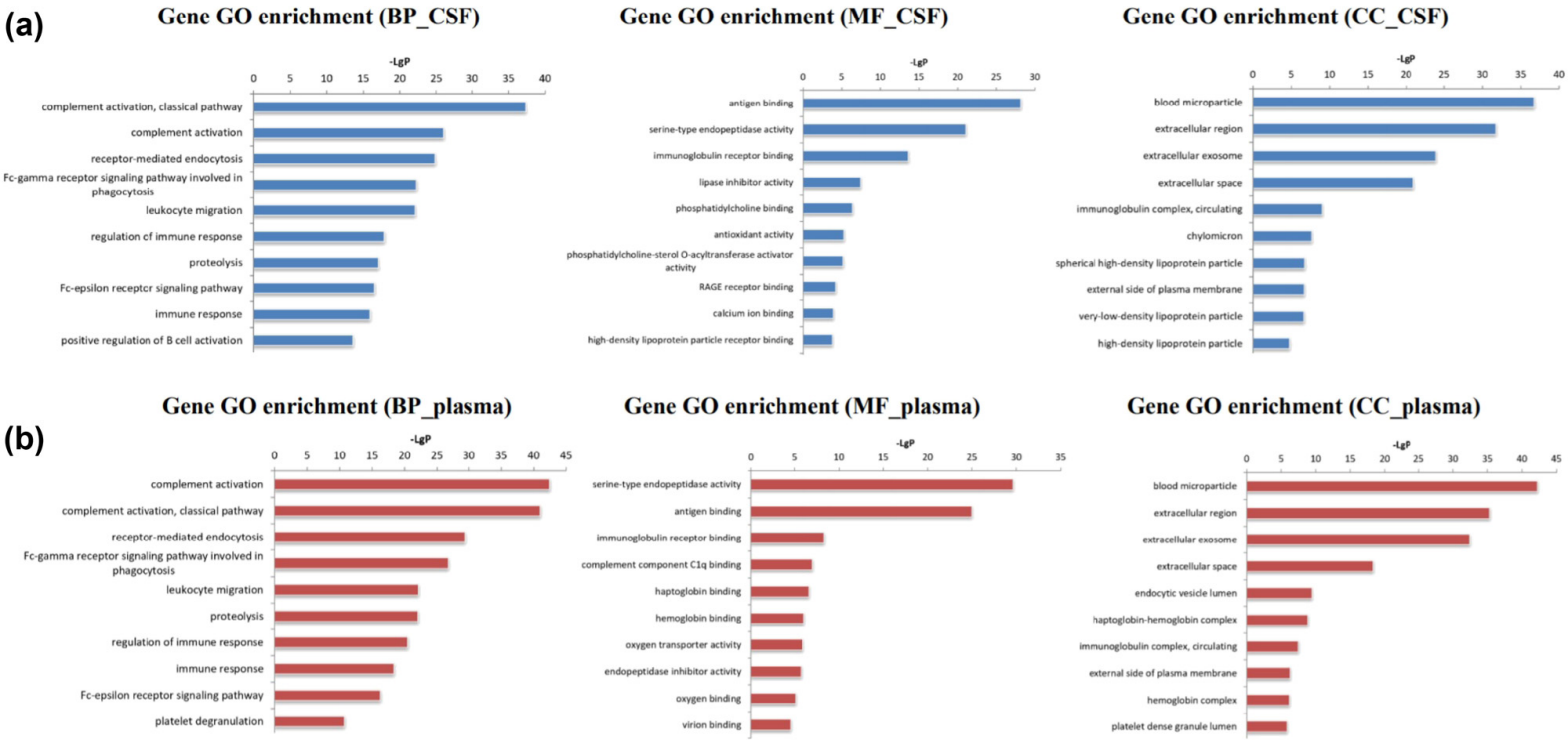
GO enrichment analysis of biological functions involving differentially expressed proteins in CSF (a) and plasma (b) of PNKD patients and controls. This figure shows the results of GO enrichment analysis of differentially expressed proteins. Differentially expressed proteins in CSF were mainly involved in blood microparticle and complex activation (classical pathway) (a), whereas those in plasma were mainly involved in blood microparticle, complex activation and complex activation (classical pathway) (b).

**Figure 5:**
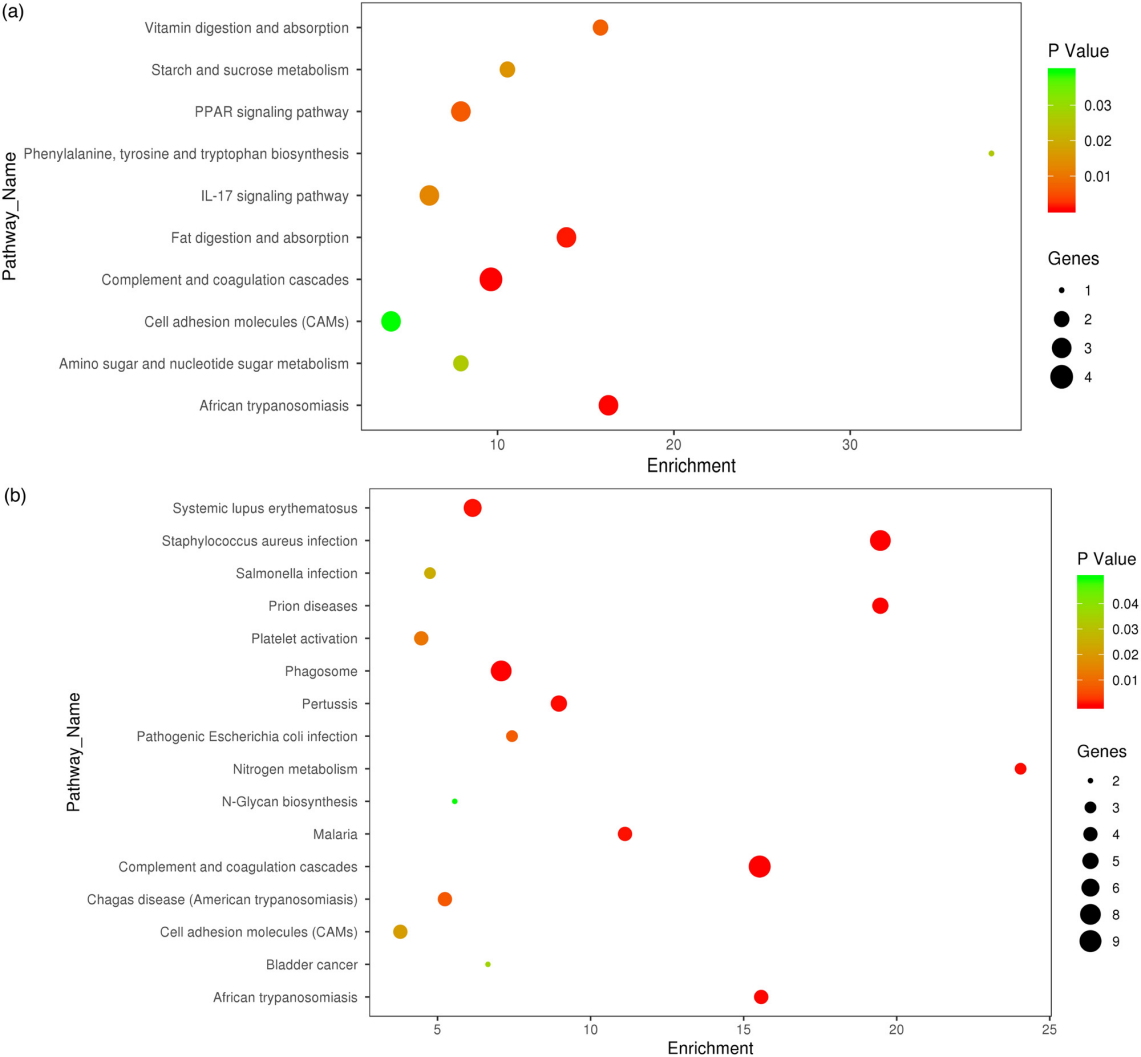
KEGG enrichment analysis of pathways involving differentially expressed proteins in CSF (a) and plasma (b) of PNKD patients and controls. This figure shows the results of GO enrichment analysis of differentially expressed proteins. The pathways with obvious enrichment of differentially expressed proteins in CSF included complex and coordination cascades and African trypanosomiasis (a), whereas the differentially expressed proteins in plasma were mainly enriched in complex and coordination cascades, *Staphylococcus aureus* infection, prion diseases, and the phasome pathway (b).

GO enrichment analysis was performed to identify biological functions involving differentially expressed proteins in CSF and plasma. The analysis results showing the top ten BPs, MFs, and CCs involving differentially expressed proteins in CSF and plasma in the form of bar charts are depicted in Figure 4. Differentially expressed proteins in CSF were mainly involved in (–LGP ≥ 35) blood microparticle and complex activation (classical pathway) (Figure 4a), whereas those in plasma were mainly involved in (–LGP ≥ 40): blood microparticle, complex activation and complex activation (classical pathway) (Figure 4b). The enrichment analysis of the KEGG signal pathway is shown in the form of a bubble diagram. The pathways with obvious enrichment of differentially expressed proteins in CSF (–LGP ≥ 3) included complex and coordination cascades and African trypanosomiasis (Figure 5a), whereas the differentially expressed proteins in plasma (–LGP ≥ 4) were mainly enriched in complex and coordination cascades, *Staphylococcus aureus* infection, prion diseases, and the phasome pathway (Figure 5b).

### 3.3 Biological network analysis of differentially expressed proteins

**Figure 6:**
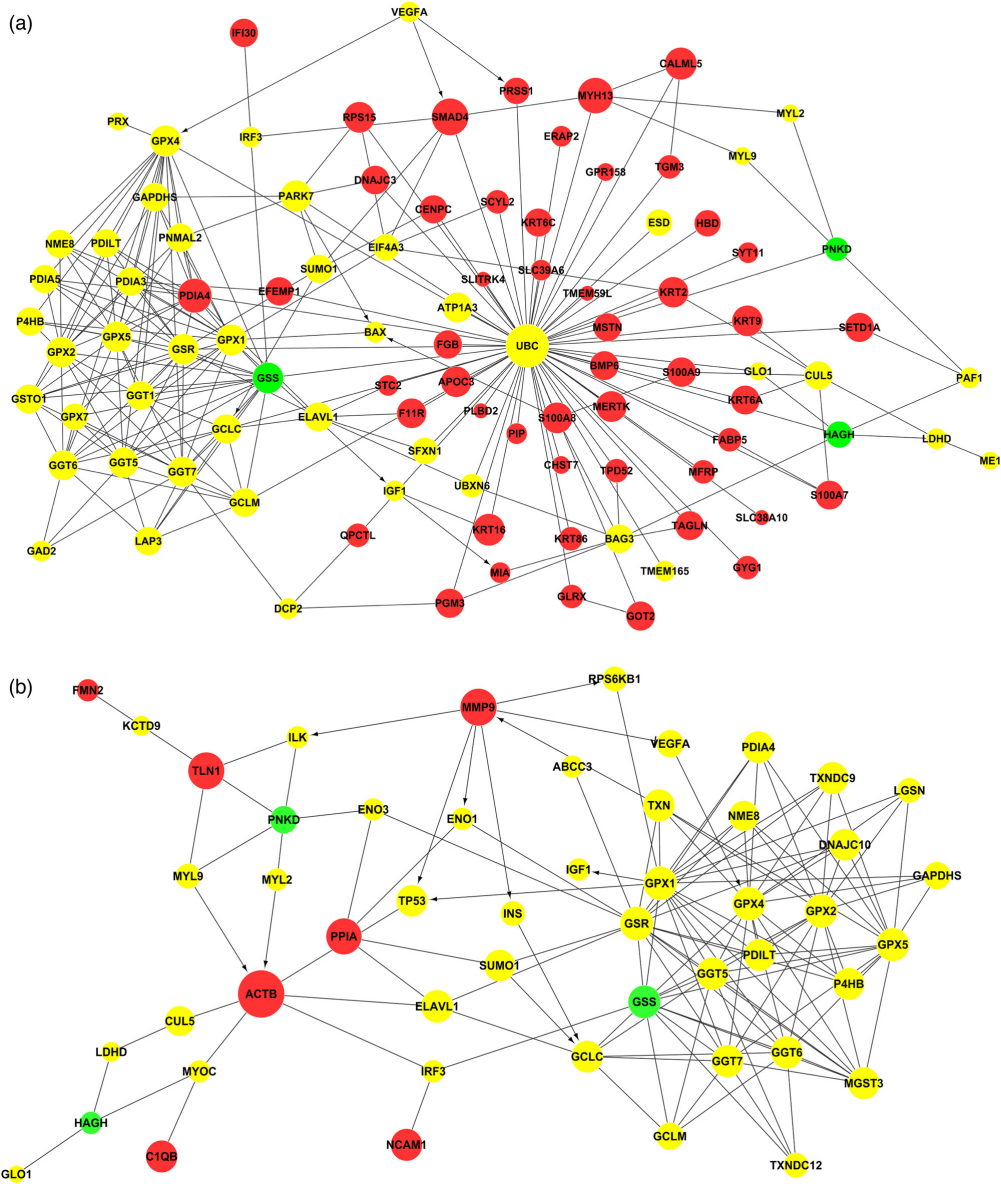
PPI network of PNKD-related proteins and differentially expressed proteins in CSF (a) and plasma (b). This figure shows the PPI network to illustrate the relationship between differentially expressed proteins and diseases in the biological network mode. In the figure, red balls represent differentially expressed proteins, green represents disease-related proteins, and yellow represents upstream and downstream signal transduction proteins connecting differentially expressed proteins/PNKD-related proteins. The size of the ball represents its betweenness centrality in the network.

By mapping differentially expressed and disease-related proteins using KEGG and STRING databases, a protein–protein interaction (PPI) network was drawn to illustrate the relationship between the differentially expressed proteins and diseases in the biological network mode. Network construction was performed using Cytoscape 3.4.0 (http://www.cytoscape.org/). In the CSF diagram, two differentially expressed proteins, recombinant mothers against decapentaplegic homolog 4 (SMAD4) and myosin heavy chain 13 (MYH13), had a high-degree relationship with other proteins (betweenness centrality >0.1) (Figure 6a). In the plasma group, two differentially expressed proteins, actin beta (ACTB) and tubulin alpha 4a (TUBA4A), showed high-degree relationships with other proteins (betweenness centrality >0.1) (Figure 6b).

## 4 Discussion

To the best of our knowledge, this study confirmed for the first time that *PNKD-L* mutation can increase the distribution of PNKD protein during subcellular localisation, both in the cytoplasm and nucleus. Ghezzi et al. [6] and others have shown that PNKD may be a mitochondrial disease. They found that *PNKD-L* was distributed in the mitochondrial membrane. All the three *PNKD* mutations reported to date have been in the N-terminal mitochondrial target sequence of *PNKD-L*, and this part is cut before *PNKD* enters the mitochondria. However, in this study, we found that PNKD protein is mainly located in the cell membrane rather than in the cytoplasm. Lee [10] found that PNKD protein was located on the cell membranes of HEK 293 cells. Similarly, Shen et al. [11,12] also reported its specific distribution on COS-7 cell membranes.

*PNKD* plays an important role in heart diseases [13], Tourette syndrome [7], and tumours [8,14], and has also been reported to play a role in regulating presynaptic extracellular secretion [14]. *PNKD-L* is specific in the brain, and its structure is similar to that of glyoxalase II, which catalyses *S*-d-lactyl-glutathione to d-lactic acid and glutathione [6]. We found that the stability of mutant PNKD-L and the content of glutathione decreased in the C6 cell line, reflecting the functional disorder and metabolic changes after the *PNKD-L* mutation. These changes could alter the catalytic effect of glyoxase II; thus, the accumulations of *S*-d-lactyl-glutathione and its upstream product, acetone aldehyde, could produce toxic reactions in neuronal cells. Furthermore, the content of acetone aldehyde could also be increased by stimulating substances such as alcohol, tea, and coffee, which also explained the mechanism of PNKD induced by such stimuli.

Proteomic technologies have been largely used to search for differentially expressed proteins, in order to find biomarkers for the diagnosis and prognosis of diseases [15,16]. The iTRAQ technique is one of the most widely used approaches because it can simultaneously analyse 8 different specimens, thus increasing throughput, while reducing experimental error [17,18,19]. In this study, we used iTRAQ labelling followed by 2D-LC-MS/MS for the quantitative proteomic analysis of CSF and plasma samples from PNKD patients and healthy controls to discover potential effective biomarkers for PNKD diagnosis. GO enrichment analysis found that blood microparticle and complex activation (classical pathway) was the common term that the differentially expressed proteins in plasma and CSF were mainly involved with. KEGG enrichment analysis indicated that the complex and coordination cascade pathway involved obvious enrichment of differentially expressed proteins in CSF and plasma. There were two differentially expressed proteins, SMAD4 and myosin heavy chain 13 (MYH13), in the CSF group, and two, ACTB and TUBA4A, in the plasma group that had a high-degree relationship with other proteins (betweenness centrality >0.1) (Figure 6). Nevertheless, SMAD4 was the only differential protein with betweenness that was related to a high GO enrichment term (complex activation [classical pathway]) and KEGG enrichment pathway (coordination cascade pathway) [20]. SMAD4 belongs to a family of signal transduction proteins that are phosphorylated and activated by transmembrane serine-threonine receptor kinases in response to transforming growth factor beta signalling via several pathways and have been reported to be biomarkers of cancers such as pancreatic cancer, juvenile polyposis syndrome, colorectal and prostate cancer, and radiation-induced lung injury [21].

There are some limitations to this study. First, the sample size of patients and healthy controls is small. Second, the relationship between SMAD4 and PNKD is unknown, and further research is needed to clarify the pathological mechanism of PNKD.

## 5 Conclusion

PNKD protein is mainly distributed on the cell membrane. *PNKD-L* mutation affects subcellular localisation, PNKD protein stability, and glyoxase II function. There were 172 and 163 differentially expressed proteins in CSF and plasma, respectively, of PNKD patients and healthy controls. For the differentially expressed proteins, blood microparticle and complex activation (classical pathway) were the common GO enrichment term, and the complex and coordination cascade pathway were the common KEGG enrichment pathway. Furthermore, SMAD4 was found to be a potential biomarker for PNKD diagnosis.

## List of abbreviations

ACTB: actin beta
CSF: cerebrospinal fluid
GO: gene ontology
iTRAQ: isobaric tags for relative and absolute quantitation
KEEG: Kyoto encyclopaedia of genes and genomes
MYH13: myosin heavy chain 13
PNKD: paroxysmal non-kinesigenic dyskinesia
PPI: protein–protein interaction
SMAD4: recombinant mothers against decapentaplegic homolog 4
TUBA4A: tubulin alpha 4a

## Supplementary Material

Supplementary Figure

## References

[1] Harvey S, King MD, Gorman KM. Paroxysmal movement disorders. Front Neurol. 2021;12:659064. 10.3389/fneur.2021.659064. PMC8232056 34177764

[2] Bago Rožanković P, Rožanković M, Romić ZČ, Bašić S. Paroxismal non-kinesigenic dyskinesia and hemidystonia associated with silent celiac disease. Clin Neurol Neurosurg. 2020;188:105586. 10.1016/j.clineuro.2019.105586. 31710883

[3] Bhatia KP. Paroxysmal dyskinesias. Mov Disord. 2011;26:1157–65. 10.1002/mds.23765. 21626559

[4] Pons R, Cuenca-León E, Miravet E, Pons M, Xaidara A, Youroukos S, et al. Paroxysmal non-kinesigenic dyskinesia due to a PNKD recurrent mutation: report of two Southern European families. Eur J Paediatr Neurol. 2012;16(1):86–9. 10.1016/j.ejpn.2011.09.008. 21962874

[5] Friedman A, Zakrzewska-Pniewska B, Domitrz I, Lee HY, Ptacek L, Kwiecinski H. Paroxysmal non-kinesigenic dyskinesia caused by the mutation of MR-1 in a large Polish kindred. Eur Neurol. 2009;61(1):39–41. 10.1159/000165348. 18948699

[6] Ghezzi D, Viscomi C, Ferlini A, Gualandi F, Mereghetti P, DeGrandis D, et al. Paroxysmal non-kinesigenic dyskinesia is caused by mutations of the MR-1 mitochondrial targeting sequence. Hum Mol Genet. 2009;18(6):1058–64. 10.1093/hmg/ddn441. 19124534

[7] Sun N, Nasello C, Deng L, Wang N, Zhang Y, Xu Z, et al. The PNKD gene is associated with tourette disorder or tic disorder in a multiplex family. Mol Psychiatry. 2018;23(6):1487–95. 10.1038/mp.2017.179. PMC5847395 28894297

[8] Wang J, Zhao W, Liu H, He H, Shao R. Myofibrillogenesis regulator 1 (MR-1): a potential therapeutic target for cancer and PNKD. J Drug Target. 2018;26(8):643–8. 10.1080/1061186X.2017.1401077. 29103325

[9] Liang S, Yu X, Zhang S, Tai J. A case of familial paroxysmal nonkinesigenic dyskinesia due to mutation of the PNKD gene in Chinese Mainland. Brain Res. 2015;1595:120–6. 10.1016/j.brainres.2014.07.047. 25107857

[10] Lee HY, Nakayama J, Xu Y, Fan X, Karouani M, Shen Y, et al. Dopamine dysregulation in a mouse model of paroxysmal nonkinesigenic dyskinesia. J Clin Invest. 2012;122(2):507–18. 10.1172/JCI58470. PMC3533379 22214848

[11] Shen Y, Lee HY, Rawson J, Ojha S, Babbitt P, Fu YH, et al. Mutations in PNKD causing paroxysmal dyskinesia alters protein cleavage and stability. Hum Mol Genet. 2011;20(12):2322–32. 10.1093/hmg/ddr125. PMC3098736 21487022

[12] Shen Y, Ge WP, Li Y, Hirano A, Lee HY, Rohlmann A, et al. Protein mutated in paroxysmal dyskinesia interacts with the active zone protein RIM and suppresses synaptic vesicle exocytosis. Proc Natl Acad Sci U S A. 2015;112(10):2935–41. 10.1073/pnas.1501364112. PMC4364199 25730884

[13] Dai W, He W, Shang G, Jiang J, Wang Y, Kong W. Gene silencing of myofibrillogenesis regulator-1 by adenovirus-delivered small interfering RNA suppresses cardiac hypertrophy induced by angiotensin II in mice. Am J Physiol Heart Circ Physiol. 2010;299(5):H1468–75. 10.1152/ajpheart.00582.2009. 20802139

[14] Wang J, Zhao W, Liu H, He H, Shao R. Myofibrillogenesis regulator 1 (MR-1): a potential therapeutic target for cancer and PNKD. J Drug Target. 2018;26(8):643–8. 10.1080/1061186X.2017.1401077. 29103325

[15] Marmor-Kollet H, Siany A, Kedersha N, Knafo N, Rivkin N, Danino YM, et al. Spatiotemporal proteomic analysis of stress granule disassembly using apex reveals regulation by SUMOylation and links to ALS pathogenesis. Mol Cell. 2020;80(5):876–91. 10.1016/j.molcel.2020.10.032. PMC7816607 33217318

[16] Saiki S, Sasazawa Y, Fujimaki M, Kamagata K, Kaga N, Taka H, et al. A metabolic profile of polyamines in parkinson disease: A promising biomarker. Ann Neurol. 2019;86(2):251–63. 10.1002/ana.25516. PMC6772170 31155745

[17] Moulder R, Bhosale SD, Goodlett DR, Lahesmaa R. Analysis of the plasma proteome using iTRAQ and TMT-based Isobaric labeling. Mass Spectrom Rev. 2018;37(5):583–606. 10.1002/mas.21550. 29120501

[18] B, urenbatu, Wang Y, Wang S, Narisu S, Wuritunashun, Gong C, et al. iTRAQ-based quantitative proteomics analysis of immune thrombocytopenia patients before and after Qishunbaolier treatment. Rapid Commun Mass Spectrom. 2021;35(3):e8993. 10.1002/rcm.8993. PMC7757159 33140498

[19] Lei D, Hong T, Li L, Chen L, Luo X, Wu Q, et al. Isobaric tags for relative and absolute quantitation-based proteomics analysis of the effect of ginger oil on bisphenol A-induced breast cancer cell proliferation. Oncol Lett. 2021;21(2):101. 10.3892/ol.2020.12362. PMC7751356 33376534

[20] Gomez-Puerto MC, Iyengar PV, García de Vinuesa A, Ten Dijke P, Sanchez-Duffhues G. Bone morphogenetic protein receptor signal transduction in human disease. J Pathol. 2019;247(1):9–20. 10.1002/path.5170. PMC6587955 30246251

[21] McCarthy AJ, Chetty R. Smad4/DPC4. J Clin Pathol. 2018;71(8):661–4. 10.1136/jclinpath-2018-205095. 29720405

